# High-Update-Rate Frequency Readout of Sinusoidal Signals for Silicon Resonant Accelerometers Using Digital Closed-Loop Frequency Tracking

**DOI:** 10.3390/mi17060683

**Published:** 2026-05-30

**Authors:** Xiangyu Zhang, Libin Huang, Song Xue, Zhenyu Sheng

**Affiliations:** 1 School of Instrument Science and Engineering, Southeast University, Nanjing 210096, China; 220233673@seu.edu.cn (X.Z.); 220243861@seu.edu.cn (S.X.); 220253855@seu.edu.cn (Z.S.); 2 Key Laboratory of Micro-Inertial Instruments and Advanced Navigation Technology, Ministry of Education, Nanjing 210096, China; 3 State Key Laboratory of Comprehensive PNT Network and Equipment Technology, Southeast University, Nanjing 210096, China

**Keywords:** silicon resonant accelerometer, sinusoidal signal readout, digital closed-loop phase-frequency locking, high-update-rate frequency tracking

## Abstract

Silicon resonant accelerometers generate sinusoidal outputs with frequency shifts that carry acceleration information. At high update rates, conventional counting-based readout suffers from gate-boundary timing quantization. This work proposes a high-update-rate frequency readout method that reconstructs frequency from the continuous phase evolution of the original sinusoidal resonant signal through quadrature demodulation, phase extraction, and phase difference rather than waveform reshaping and edge counting. To implement the proposed readout chain, an FLL–PLL cooperative loop was included to assist coarse acquisition and fine tracking on a Zynq-7020 platform. This study focuses on the readout principle, FPGA implementation, and prototype-level evaluation. At a 1 kHz update rate, the proposed method showed a lower theoretical quantization limit than the synchronous multi-cycle counting method. Under room-temperature conditions, after a 30 min startup, the proposed method reduced the standard deviation of the 1-second-averaged zero-bias output over 1800–5400 s from 4.1 μg to 2.4 μg and reduced the frequency-difference peak-to-peak value from 0.03743 Hz to 0.02410 Hz. These results support the feasibility and practical value of the proposed method for high-update-rate readout of sinusoidal resonant signals under the tested steady-state conditions.

## 1. Introduction

Silicon resonant accelerometers have attracted sustained interest for high-performance inertial sensing because the measurand is converted into a frequency shift, which is advantageous for digital readout and long-term stability [1,2,3,4]. In such devices, the final output quality depends not only on the mechanical performance of the resonant structure but also on the accuracy, stability, and update rate of the frequency readout chain. Beyond resonator design, the readout method is a key factor in determining the practical resolution and dynamic tracking capability of the sensor system.

Existing studies have shown that improvements in resonator topology, packaging, synchronization, oscillation control, and interface circuitry can enhance the overall performance of resonant accelerometers [2,3,4,5,6,7,8,9]. However, these advances do not by themselves remove the readout bottleneck when a high output update rate is required. In particular, for resonant sensors with outputs that are inherently sinusoidal, the frequency readout stage must balance three competing requirements: high update rate, low equivalent quantization error, and reliable real-time tracking under practical hardware constraints.

This challenge is difficult to address using conventional counter-based frequency measurement alone. Direct counting and gated counting methods estimate frequency by counting signal edges within a finite observation interval, and their effective quantization error increases as the interval is shortened [10]. This limitation becomes more pronounced at kHz-level output update rates, where only a limited number of cycles are observed in each update window. Moreover, when a sinusoidal resonant signal is reshaped into a pulse train for edge counting, part of the continuous phase information carried by the original waveform is discarded. Therefore, estimating frequency from phase evolution rather than from integer-cycle counting is more suitable for high-update-rate readout of resonant sinusoidal signals [10,11,12,13].

Although phase-based estimation and digital tracking techniques have been widely studied [11,12,13], their implementation-oriented treatment for high-update-rate resonant-signal readout remains insufficiently discussed. Existing studies tend to focus on the phase-based principle or on loop-tracking concepts themselves, whereas for FPGA-oriented real-time readout chains, several practical issues must be resolved simultaneously, including I/Q synchronization, valid-sample alignment, state switching, synchronous DDS frequency-word updating, and fixed-point numerical constraints. These implementation-level issues are critical to whether a high-update-rate phase-based readout scheme can be reliably translated into a reproducible hardware chain.

Accordingly, this study develops a high-update-rate FPGA frequency readout method for silicon resonant accelerometer signals. The main contributions are threefold. First, a phase-difference-based frequency readout framework for sinusoidal resonant signals is established, and its error-transfer mechanism is clarified to show how its dominant limitation differs from that of conventional counting-based methods. Second, an FPGA readout chain is implemented with synchronized I/Q processing, valid-sample alignment, fixed-point realization, and synchronous DDS frequency-word updating to maintain timing consistency in real-time operation. In this implementation, an FLL–PLL-based cooperative loop structure is included to support acquisition and steady-state tracking. Third, the proposed method is evaluated through theoretical analysis, hardware implementation on a Zynq-7020 core board (ALIENTEK, Guangzhou Xingyi Electronic Technology Co., Ltd., Guangzhou, China), and prototype-level experiments on a silicon resonant accelerometer test platform under room-temperature conditions.

## 2. Proposed Readout Method and System Implementation

### 2.1. Overall Architecture of the Frequency Readout System

The prototype silicon resonant accelerometer used in this work produces sinusoidal outputs from the upper and lower resonant beams, and the corresponding frequency variations encode the applied acceleration. To support high-update-rate readout, the backend system was designed to estimate frequency directly from phase evolution rather than from square-wave reshaping and edge counting. Accordingly, the overall architecture integrates synchronous acquisition, FPGA-based phase-difference processing, closed-loop frequency tracking, and host-computer data output.

The complete readout chain was implemented on a Xilinx Zynq–7020 platform as the backend digital readout module of the prototype accelerometer system. The programmable logic (PL) was used for real-time signal processing and closed-loop tracking tasks, while the processing system (PS) was used for auxiliary functions such as parameter configuration, state monitoring, and communication with the host computer. This partition was adopted to preserve timing determinism in the core readout path while retaining implementation flexibility for system control and data management.

**Figure 1:**
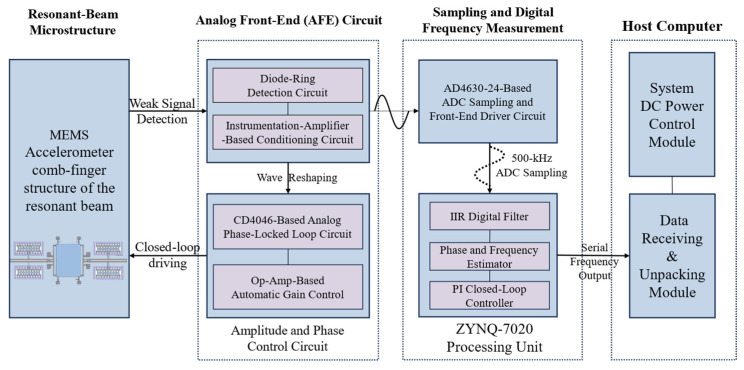
Overall architecture of the prototype silicon resonant accelerometer frequency readout system.

Figure 1 shows the overall architecture of the proposed frequency readout system. The frontend closed-loop drive circuit is used to establish and maintain stable oscillation of the resonant elements. The analog outputs of the upper and lower resonant beams are first conditioned by the frontend amplification and differential driving circuits, and they are then delivered to the analog-to-digital converter (ADC). The ADC performs synchronous dual-channel sampling so that the subsequent digital processing is based on temporally aligned data. After digitization, the sampled data are transferred to the FPGA for real-time frequency readout.

Within the FPGA, the digital readout chain consists of five stages: NCO/DDS-based generation of orthogonal reference signals, quadrature mixing of the sampled resonant signal, low-pass filtering and decimation of the I/Q components, phase extraction and phase-increment calculation for frequency estimation, and feedback of the estimated frequency to the NCO/DDS to realize closed-loop tracking.

Rather than a simple cascade of independent DSP modules, the proposed architecture is organized around a unified valid-sample boundary. The two ADC channels are sampled synchronously, the I/Q demodulation branches share the same sampling-valid event, and only simultaneously valid filtered I/Q outputs are passed to the decimation and phase-extraction stages. As a result, phase difference, loop-state updating, and DDS frequency-word refreshing are all referenced to the same decimated valid boundary, which suppresses artificial phase error caused by I/Q timing mismatch and ensures that each closed-loop frequency update is associated with a well-defined phase observation.

An output path is further included for real-time transmission of the measured frequency data to the host computer through an RS485-to-USB link, enabling data visualization, storage, and offline analysis. In the implemented prototype, the upper and lower resonant-beam outputs were processed by two parallel readout channels with the same phase-difference-based estimation procedure. The accelerometer output used in the subsequent scale-factor and zero-bias evaluations was then formed from the difference between the two tracked resonant frequencies. Overall, the architecture provides an integrated platform for synchronous acquisition, phase-based frequency readout, closed-loop tracking, and experimental validation of the prototype accelerometer system.

### 2.2. Principle of Phase-Difference Frequency Estimation

**Figure 2:**
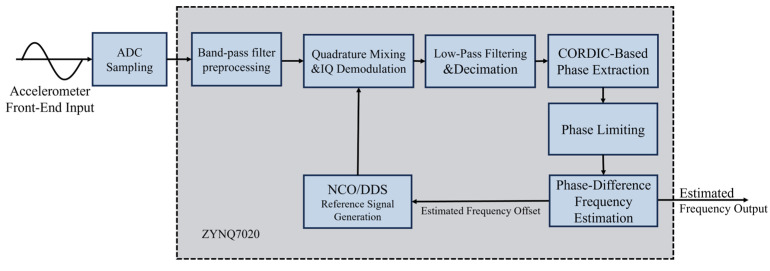
Principle of digital closed-loop frequency tracking for the sinusoidal resonant signal.

The sinusoidal output of a silicon resonant accelerometer carries the frequency variation induced by the applied acceleration. Rather than converting this signal into a square wave for edge counting, the proposed method estimates frequency directly from phase evolution. Accordingly, the frequency readout problem is reformulated as phase-rate estimation and is implemented through quadrature demodulation, phase extraction, and phase difference. The corresponding digital closed-loop frequency-tracking scheme for the sinusoidal resonant signal is illustrated in Figure 2.

Let the silicon resonant accelerometer output be expressed as

(1) $$x(t)=A\cos\left(2\pi \int_{0}^{t}f_{in}\left(\tau \right)d\tau +\phi_{0}\right)=A\cos\phi (t)$$

where *A* is the signal amplitude, ϕ(t) is the instantaneous phase, and $\phi_{0}$ is the initial phase. The instantaneous frequency is related to the phase by

(2) $$f_{in}(t)=\frac{1}{2\pi }\cdot \frac{d\phi (t)}{dt}$$

Equation (2) shows that the signal frequency can be obtained once the continuous phase variation is accurately tracked. After anti-aliasing filtering and analog-to-digital conversion, the sampled input signal can be written as

(3) $$x[n]=A\cos\left(2\pi \frac{f_{in}}{F_{s}}n+\phi_{0}\right)$$

where $f_{in}$ is the input frequency, and $F_{s}$ is the sampling frequency.

A numerically controlled oscillator (NCO) is used to generate orthogonal reference signals at frequency $f_{NCO}$. By mixing the sampled input with the cosine and sine references and then applying low-pass filtering, the high-frequency sum terms are removed, and only the low-frequency difference terms are retained. 

We define the residual frequency offset as

(4) $$\Delta f=f_{in}-f_{\mathrm{NCO}}$$

The in-phase and quadrature components after quadrature demodulation can be expressed as

(5) $$I[n]=\frac{A_{I}}{2}\cos\left(2\pi \frac{\Delta f}{F_{s}}n+\Delta \phi_{0}\right),Q[n]=\frac{A_{Q}}{2}\sin\left(2\pi \frac{\Delta f}{F_{s}}n+\Delta \phi_{0}\right)$$

where $\Delta \phi_{0}$ is the initial phase difference between the input signal and the NCO reference. After quadrature demodulation, the original carrier-frequency component is translated into a low-frequency residual phase term, and the frequency information is therefore represented by the phase evolution of the demodulated I/Q signals. 

The instantaneous residual phase is then obtained from the demodulated (*I*) and (*Q*) signals using the four-quadrant arctangent operation:

(6) $$\hat{\theta }[k]=\mathrm{atan}2\left(Q[k],I[k]\right)$$

where *k* denotes the sample index after decimation. In the hardware implementation, the arctangent operation is realized by a CORDIC (Coordinate Rotation Digital Computer) IP core to achieve a good trade-off between computational efficiency and real-time performance [14,15].

Since the arctangent output is confined to the principal interval (–π, π], a direct difference of θ[k] may produce discontinuities when the phase crosses the ±π boundary. Therefore, phase unwrapping is performed on the phase increment rather than on the absolute phase. The wrapped phase increment is defined as

(7) $$\Delta \hat{\theta }[k]=wrap\left(\hat{\theta }[k]-\hat{\theta }[k-1]\right)$$

where wrap(.) maps the phase difference back to (–π, π]. Accordingly,

(8) $$wrap\left(x\right)=\left\{\begin{array}{c} x-2\pi ,x>\pi \\ x+2\pi ,x\leq -\pi \\ x,otherwise \end{array}\right.$$

Jitter near the ±π boundary is further reduced by band-limited I/Q preprocessing, while loop-state decisions are based on the wrapped phase increment and the smoothed residual-frequency error, preventing isolated phase perturbations from directly switching states or updating the DDS word. If the update rate after decimation is *F_u_*, the residual frequency offset can be estimated from the phase increment as

(9) $$\hat{\Delta f}[k]=\frac{F_{u}}{2\pi }\cdot \Delta \hat{\theta }[k]$$

The measured input frequency is then reconstructed as

(10) $${\hat{f}}_{in}[k]=f_{\mathrm{NCO}}[k]+\hat{\Delta f}[k].$$

Therefore, the proposed method reconstructs the input frequency from the phase increment between the measured signal and the NCO reference. Unlike conventional gated counting and waveform-reshaping-based readout approaches, it estimates frequency directly from the continuous phase evolution of the original sinusoidal signal rather than from integer-cycle accumulation within a finite gate window, thereby avoiding the gate-boundary ±1-count uncertainty mechanism.

The above phase-difference formulation also clarifies the error-transfer mechanism of the proposed method. Let the extracted phase after decimation be written as

(11) $$\hat{\theta }[k]=\theta_{\mathrm{true}}[k]+\varepsilon_{\phi }[k]$$

where $\theta_{\mathrm{true}}[k]$ is the true residual phase, and $\varepsilon_{\phi }[k]$ denotes the phase-extraction error. When the residual phase increment remains within the principal interval and no wrap folding occurs, the estimated phase increment can be expressed as

(12) $$\Delta \hat{\theta }[k]=\Delta \theta_{\mathrm{true}}[k]+\varepsilon_{\phi }[k]-\varepsilon_{\phi }[k-1]$$

Accordingly, the residual-frequency estimation error is

(13) $$e_{f}[k]=\frac{F_{u}}{2\pi }\cdot \left(\varepsilon_{\phi }[k]-\varepsilon_{\phi }[k-1]\right)$$

where *F_u_* is the update rate after decimation. This relation indicates that the frequency-estimation error of the proposed method is determined by the phase-extraction error rather than by integer-cycle counting uncertainty. Therefore, the advantage of the proposed phase-difference method does not lie in eliminating frequency-estimation error, but in converting the dominant error mechanism under high-update-rate operation from the gate-boundary ±1-count uncertainty of conventional counters to the resolution and noise performance of the phase-extraction chain [10,11].

If the phase-extraction error is modeled as a zero-mean random process with variance $\sigma_{\phi }^{2}$, the corresponding frequency-error variance is proportional to 1/T_u^2^_. This indicates that, under a fixed phase-noise level, increasing the update rate increases the sensitivity of frequency estimation to phase error. In other words, the proposed method does not remove the error source itself; rather, it changes the dominant limitation from counter quantization at the gate boundary to phase-resolution, phase-noise, and implementation-related errors in the phase-extraction chain.

For quantization-limited analysis, the phase word used in the loop computation is represented in a Q16.16 radian-scaled format. Therefore, the minimum phase increment is

(14) $$\Delta \theta_{\min}=2^{-16}\;\mathrm{rad}$$

According to the phase-increment-to-frequency relation in Equation (9), the corresponding single-update equivalent frequency quantization scale is

(15) $$\Delta f_{\min}\approx \frac{F_{u}}{2\pi }\Delta \theta_{\min}=\frac{F_{u}}{2\pi \cdot 2^{16}}$$

where $F_{u}\;$ is the update rate after decimation. This expression represents only the quantization-limited frequency floor associated with phase resolution. It should be noted that the 16-bit value used here refers to the 16 fractional bits of the Q16.16 radian-scaled phase word used in the loop computation rather than to a 16-bit phase code uniformly covering the full 2π interval. In the actual FPGA implementation, the CORDIC IP core is configured with a 32-bit phase output, as described in Section 2.5. The equivalent 16-bit figure adopted here is a conservative analytical reference introduced solely for comparison purposes and should not be interpreted as the hardware phase resolution.

For comparison, the synchronous multi-cycle counting method aligns the effective gate with the measured-signal edges. Under this mechanism, the dominant quantization uncertainty is determined by the reference-clock count. The corresponding quantization-limited frequency resolution can be approximated as

(16) $$\Delta f_{\mathrm{sync}}\approx \frac{f_{in}}{f_{\mathrm{clk}}}\cdot F_{u}$$

where *f_in_* is the frequency of the measured signal, *F_u_* is the output update rate, and *f_clk_* is the reference-clock frequency. Thus, although the synchronous multi-cycle counting method improves upon direct counting, its quantization floor still increases linearly with the update rate. By contrast, the dominant limitation of the proposed digital closed-loop frequency tracking method is transferred to the phase-extraction chain. Therefore, under high-update-rate conditions and with sufficient effective phase resolution, digital closed-loop frequency tracking can provide a lower theoretical quantization floor for sinusoidal resonant-signal readout than conventional gated counting [13]. This comparison is intended to contrast the dominant quantization mechanisms of the two readout strategies and should not be interpreted as a full prediction of the measured prototype error.

### 2.3. Closed-Loop Cooperation and State-Switching Mechanism

**Figure 3:**
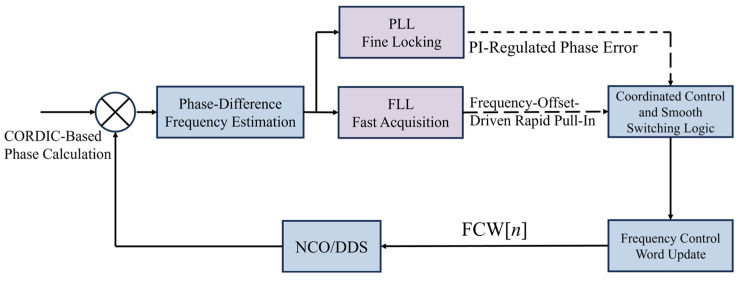
Collaborative control architecture of the FLL–PLL frequency tracking scheme.

To achieve both large-offset frequency acquisition and fine steady-state tracking, a cooperative closed-loop architecture combining a frequency-locked loop (FLL) and a phase-locked loop (PLL) was implemented. All loop operations were referenced to the same valid decimated I/Q sample boundary. In the present implementation, the ADC sampling rate was 500 kHz, and the decimation factor was 500, resulting in a 1 kHz loop-update rate. At each valid update instant, one complete loop cycle was executed, including residual-frequency estimation, error smoothing, state evaluation, command generation, and DDS frequency-control-word refreshing. The corresponding cooperative control architecture of the FLL–PLL frequency-tracking scheme is shown in Figure 3.

The FLL path was used for coarse frequency acquisition. After phase extraction and phase difference, a residual-frequency estimate Δ*f*_est_[*n*] was obtained as described in Section 2.2. To suppress false state transitions caused by sample-to-sample jitter, the state machine used a smoothed residual-frequency error Δ*f*_sm_[*n*] rather than the instantaneous estimate according to (17):

(17) $$\Delta f_{\mathrm{sm}}[n]=\alpha \cdot \Delta f_{\mathrm{sm}}[n-1]+(1-\alpha )\cdot \Delta f_{\mathrm{est}}[n]$$

where *α* is the smoothing coefficient. All state-transition thresholds defined below were applied to Δ*f*_sm_[*n*].

The PLL path was introduced for fine tracking after coarse frequency alignment had been established. Because the absolute phase output of the arctangent block contains an arbitrary initial phase offset, lock assessment was based on a relative phase error referenced to the phase captured at lock entry. Let *θ*[*n*] denote the extracted phase and *θ*_ref_ denote the reference phase stored when the loop enters the LOCK state. The PLL phase error was defined as

(18) $$e_{\phi }[n]=\mathrm{wrap}\left(\theta [n]-\theta_{\mathrm{ref}}\right)$$

After the smoothed residual-frequency error and the relative phase error were obtained, the FLL and PLL commands were generated in discrete time. The FLL was used to integrate the smoothed residual-frequency error for coarse correction, whereas the PLL provided fine adjustment through a PI-type law driven by the relative phase error.

(19) $$u_{\mathrm{FLL}}[n]=u_{\mathrm{FLL}}[n-1]+K_{\mathrm{FLL}}\cdot \Delta f_{\mathrm{sm}}[n]$$

(20) $$u_{\mathrm{PLL}}[n]=K_{p}\cdot \frac{F_{u}}{2\pi }e_{\phi }[n]+K_{i}\cdot \sum_{i=0}^{n}\frac{F_{u}}{2\pi }e_{\phi }[i]$$

In the hardware implementation, the PLL bandwidth was determined from the discrete-time PI control law in Equation (20) rather than from the proportional term alone. The 1 kHz update rate defines the discrete-time phase-observation and DDS-refresh interval, while the effective closed-loop bandwidth is set by the selected PI gains and the state-dependent scaling applied to the PLL correction term. To clarify this relationship, the normalized PLL correction can be expressed as

(21) $$\Omega_{\mathrm{PLL},s}[n]=\frac{2\pi }{F_{u}}u_{\mathrm{PLL},s}[n]=K_{p,s}e_{\phi }[n]+K_{i,s}\sum_{i=0}^{n}e_{\phi }[i]$$

where *s* denotes the PULL_IN or LOCK state. Considering the weighting factor in the combined control command, the effective PI gains become

(22) $$K_{p,\mathrm{eff},s}=\beta_{s}K_{p,s}\;\;\;\;\;\;\;\;\;\;K_{i,\mathrm{eff},s}=\beta_{s}K_{i,s}$$

Therefore, the equivalent PLL bandwidth is a design result of the discrete-time PI parameters under the 1 kHz update rate. In the PULL_IN state, a relatively higher effective bandwidth is used to improve residual-frequency tracking and relocking after coarse FLL acquisition. In the LOCK state, a lower effective bandwidth is used to suppress phase-extraction noise and fixed-point perturbations during steady fine tracking. This state-dependent bandwidth design allows the loop to balance dynamic tracking capability and steady-state readout stability.

A three-state finite-state machine, consisting of SEARCH, PULL-IN, and LOCK, was used to coordinate the FLL and PLL actions.

In the SEARCH state, only the FLL correction was applied to provide robust coarse acquisition under a large initial frequency mismatch. Transitions from SEARCH to PULL–IN were allowed only when two conditions were simultaneously satisfied: |Δ*f*_sm_[*n*]| < T_PI_ and the SEARCH dwell counter had reached the minimum dwell count N_SEARCH_. Upon entering the PULL–IN state, the SEARCH dwell counter was cleared. In the PULL–IN state, the FLL remained dominant, while a weighted PLL correction was introduced. Transition from PULL–IN to LOCK was enabled only when |Δ*f*_sm_[*n*]| < T_L_ for N_LOCK_ consecutive update cycles. The LOCK confirmation counter was reset whenever this condition was violated. Conversely, if |Δ*f*_sm_[*n*]| > T_S_ during PULL–IN, the loop returned to SEARCH. If |Δ*f*_sm_[*n*]| > T_U_ in LOCK, the loop returns to PULL–IN. When the state first transitioned from PULL–IN to LOCK at update instant n, the coarse FLL command and the reference phase at the same instant were latched as u_hold_ = u_FLL_[n] and *θ*_ref_ = *θ*[*n*], respectively, and the PLL path thereafter provided fine adjustment around this held coarse command.

**Figure 4:**
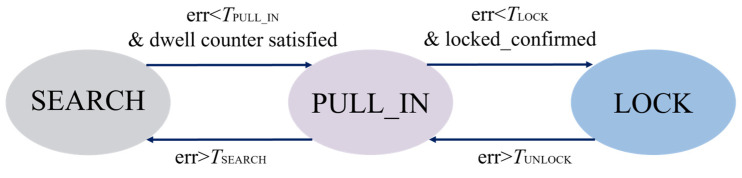
State transition diagram of the frequency tracking loop.

As shown in Figure 4, after state evaluation at each valid update instant, the control command was generated according to the resulting loop state. Let u_FLL_ [n] denote the coarse correction generated by the FLL path, let u_PLL_[n] denote the PLL correction, and let u_hold_ denote the held coarse correction recorded at lock entry. The combined control command was defined as

(23) $$u[n]=\left\{\begin{array}{ll} u_{\mathrm{FLL}}[n], & \mathrm{SEARCH},\\ u_{\mathrm{FLL}}[n]+\beta_{\mathrm{PI}}\cdot u_{\mathrm{PLL}}[n], & \mathrm{PULL}\_\mathrm{IN},\\ u_{\mathrm{hold}}[n]+\beta_{L}\cdot u_{\mathrm{PLL}}[n], & \mathrm{LOCK}, \end{array}\right.$$

where β_PI_ and β_L_ are the PLL weighting factors in the PULL–IN and LOCK states, respectively. In the reported implementation, both weighting factors were fixed at 0.5. After command generation, the DDS frequency control word was refreshed synchronously. In this way, phase difference, state switching, and frequency-word updating were all referenced to the same decimated valid boundary. Since the control command was generated and refreshed at every valid update instant in all three states, the frequency output was not interrupted during the FLL-to-PLL transition. In the SEARCH and PULL_IN states, the output represents the acquisition-stage tracking result, while in the LOCK state, it corresponds to the steady fine-tracking result used for high-stability frequency readout. The main loop-cooperation and state-switching parameters used in the reported experiments are summarized in Table 1.

### 2.4. Hardware Implementation

The proposed readout hardware was developed as the backend measurement module of the prototype silicon resonant accelerometer system. The design emphasis was placed on synchronous dual-channel acquisition, low-noise analog interfacing, and deterministic real-time FPGA processing so that the subsequent phase-difference estimation and FLL–PLL tracking could be executed on a stable hardware basis.

To satisfy the requirements of frontend bandwidth, measurement accuracy, and dual-channel synchronous real-time sampling, the AD4630-24 SAR ADC (Analog Devices, Inc., Wilmington, MA, USA) was selected as the data-acquisition device. For the approximately 33 kHz sinusoidal outputs of the resonant beams, the high signal-to-noise ratio and effective resolution of the AD4630-24 help reduce the influence of quantization noise on the readout signal. In addition, the device supports dual-channel simultaneous sampling, enabling the upper and lower resonant-beam signals to be acquired at the same instant, which is beneficial for suppressing common-mode errors induced by reference-clock jitter and drift in shared signal paths. Its Flexi-SPI interface also provides sufficient bandwidth for real-time frequency measurement and data transfer.

**Figure 5:**
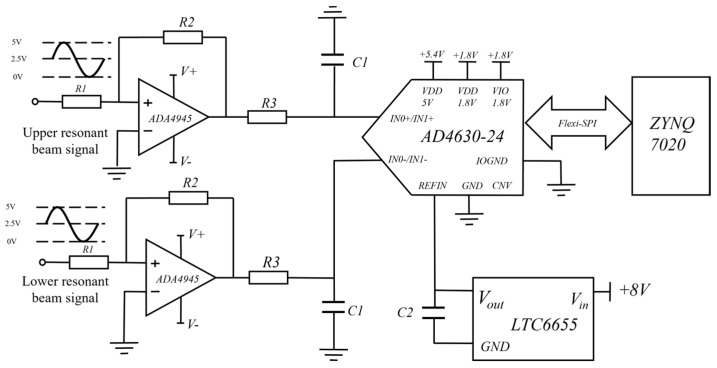
Schematic diagram of the ADC sampling and driver circuit.

To match the ADC input requirements, as shown in Figure 5, an ADA4945-1 differential driver (Analog Devices, Inc., Wilmington, MA, USA) differential driver was used in the analog frontend to perform single-ended-to-differential conversion and improve common-mode noise rejection. Its output common-mode level was adjusted to match the ADC input range, thereby reducing distortion and improving full-scale utilization for the bipolar resonant signal. Owing to its high bandwidth, low noise, and low output impedance, the driver was well suited to the ADC sampling network.

For the power subsystem, an LTC6655 precision voltage reference (Analog Devices, Inc., Wilmington, MA, USA) was adopted as a low-noise reference source for the ADC, and an LT3045 low-noise LDO (Analog Devices, Inc., Wilmington, MA, USA) low-noise LDO with local RC filtering was used to provide clean analog power. In addition, ferrite-bead isolation and local decoupling were applied at critical analog nodes to suppress coupled noise from the supply, ground return, and reference paths, thereby improving the stability of the acquisition chain.

Because the phase-difference-based frequency measurement method is sensitive to amplitude consistency, phase consistency, sampling synchronism, and frontend noise performance, the hardware design serves not only for signal acquisition but also as a low-noise and dual-channel synchronous interface foundation for subsequent phase extraction and closed-loop frequency estimation.

### 2.5. Digital Filter and Key Implementation Parameters

To support stable phase extraction and closed-loop tracking, digital filtering was incorporated into the FPGA readout chain. In the present implementation, the most critical filtering stage was the post-demodulation low-pass filter, which suppresses high-frequency sum components, retains the residual phase information, and enables synchronous decimation before phase extraction and loop updating. 

After quadrature demodulation, matched low-pass filters were applied to the I and Q branches to suppress the high-frequency sum components and retain residual phase information. In the present implementation, a sixth-order Butterworth IIR low-pass filter with a cutoff frequency of 2 kHz was adopted. Compared with an FIR filter providing similar selectivity, the IIR structure requires lower computational complexity and fewer hardware resources, which is advantageous for real-time FPGA implementation. To improve numerical robustness in fixed-point realization, the sixth-order filter was implemented as three cascaded second-order sections (SOSs) rather than as a single direct high-order form. The filter response was designed and verified in MATLAB R2023a before hardware deployment.

In the hardware implementation, each SOS followed the state-update form

(24) $$d[n]=x[n]+a_{1}d[n-1]+a_{2}d[n-2]$$

and the corresponding output was computed as

(25) $$y[n]=b_{0}d[n]+b_{1}d[n-1]+b_{2}d[n-2]$$

Accordingly, the coefficients listed in Table 2 are those actually used in the Verilog implementation of the SOS IIR filter rather than ideal floating-point reference values. The input and output data paths were represented as 24-bit signed quantities. To preserve sufficient internal precision during multiplication and accumulation, the arithmetic path employed 54-bit signed operands and 108-bit intermediate products. In hardware, the multiplication was realized using a Xilinx Multiplier IP core(12.0), and the internal result after the third SOS stage was truncated to a 24-bit signed output for subsequent decimation and phase extraction.

After low-pass filtering, the synchronously filtered I/Q data were decimated by a factor of 500, reducing the data rate from 500 kHz to 1 kHz for phase extraction, loop-state updating, and DDS frequency-control-word refreshing. In the implemented timing scheme, only simultaneously valid filtered I/Q outputs were admitted into the subsequent stages so that the decimated phase observations remained aligned with the synchronized update logic described above.

A frontend band-pass preprocessing stage was also included to improve spectral purity near the resonant band. Because the analog drive and frontend circuitry may introduce out-of-band interference and second-harmonic spurs, a sixth-order Butterworth IIR band-pass filter was placed after ADC sampling. With the ADC operating at 500 kHz, the passband was set to 30.5–35.5 kHz to match the nominal resonant frequency near 33 kHz while suppressing out-of-band components.

The key implementation parameters of the proposed readout system are summarized in Table 3. In the present design, the numerically controlled oscillator was implemented using the Xilinx DDS Compiler IP core(6.0), operated at 50 MHz with a 48-bit phase accumulator and 16-bit sine/cosine outputs. Phase extraction was realized using the Xilinx CORDIC IP core configured for Arctan operation, with 24-bit signed-fraction inputs, 32-bit phase outputs, and a latency of 36 clock cycles. The CORDIC parameters were selected by balancing ADC resolution, fixed-point quantization error, FPGA resource usage, and timing requirements. Specifically, the 24-bit I/Q input matches the preceding data path, the 32-bit phase output reduces phase quantization error, and the 36-clock-cycle latency is much shorter than the 1 ms loop-update interval and is compensated by valid-signal alignment in the FPGA implementation. Together with the low-pass filtering, decimation chain, and fixed-point SOS implementation, these parameter settings provide a reproducible numerical and timing basis for the proposed FPGA phase-difference readout system.

## 3. Results

### 3.1. Theoretical Quantization-Limited Comparison of Frequency Readout Methods

**Figure 6:**
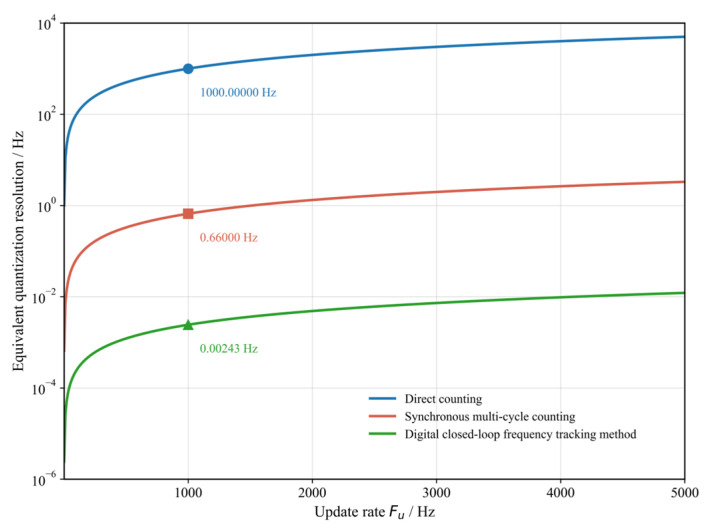
Theoretical quantization-limited resolution of direct counting, synchronous multi-cycle counting, and digital closed-loop frequency tracking method versus update rate.

Figure 6 compares the theoretical quantization-limited resolution of direct counting, synchronous multi-cycle counting, and the proposed digital closed-loop frequency tracking method as a function of the update rate. Both axes are plotted on logarithmic scales to facilitate comparisons over a wide dynamic range. As the update rate increases, the quantization-limited resolution of all three methods deteriorates but to markedly different extents. At an update rate of 1 kHz, the corresponding theoretical limits are 1000 Hz, 0.660 Hz, and 0.00243 Hz, respectively. The value of 0.00243 Hz for the digital closed-loop frequency tracking method is calculated from the Q16.16 radian-scaled phase word used in the loop computation. 

This result suggests that phase-difference estimation is better suited than conventional counting-based methods for high-update-rate readout of sinusoidal resonant signals. Figure 6 reflects only the dominant quantization mechanisms of the three methods and does not represent the full measured error of the implemented prototype, which is also affected by phase noise, circuit noise, fixed-point effects, and clock stability.

### 3.2. Closed-Loop Tracking Simulation Results

**Figure 7:**
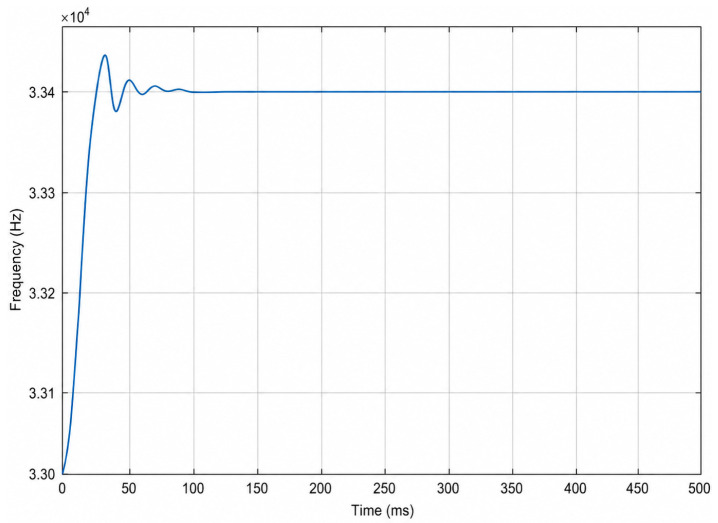
Simulated closed-loop frequency tracking under a 400 Hz initial offset using Simulink MATLAB R2023a and Simulink.

The closed-loop dynamic behavior of the proposed method was evaluated using MATLAB R2023a and Simulink. The simulation was implemented as a discrete-time behavioral model with the same update rate, loop coefficients, state-switching thresholds, and DDS frequency-word update logic as those used in the FPGA implementation. As shown in Figure 7, the initial NCO frequency was set to 33,000 Hz, and the input sinusoidal frequency was set to 33,400 Hz, corresponding to an initial offset of 400 Hz. Under this condition, the output frequency rapidly converged toward the target value and entered a stable tracking region within approximately 100 ms. The transient response exhibited a clear two-stage behavior, in which the FLL dominated the large-offset pull-in stage, and the PLL progressively refined the residual error in the near-lock region. This simulation verifies the state-transition logic and closed-loop convergence behavior. Measurement noise, phase noise, ADC quantization noise, and frontend circuit noise were not included in this behavioral model.

### 3.3. Prototype Accelerometer Test Platform and Scale-Factor Evaluation

**Figure 8:**
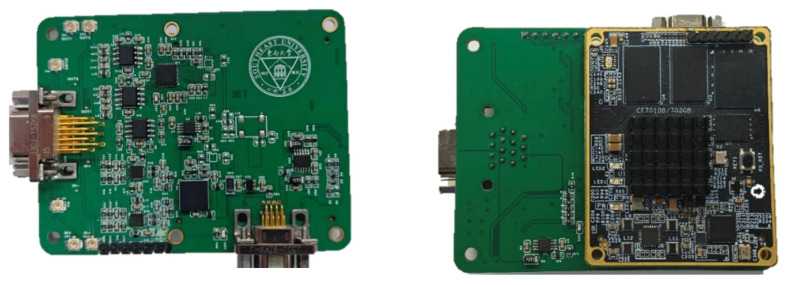
Photograph of the Zynq-7020-based frequency-readout board.

After the method-level verification, experiments were conducted on a prototype silicon resonant accelerometer developed by the research group. The prototype included the resonant sensor head, the frontend drive and signal-conditioning circuits, and a Zynq-7020-based frequency-readout board, as shown in Figure 8. The upper and lower resonant-beam sinusoidal outputs were delivered to the FPGA readout chain for synchronous acquisition and real-time processing, and the measured data were transmitted to the host computer through an RS485 interface for storage and offline analysis. This platform was used to evaluate the room-temperature scale-factor characteristics and zero-bias stability achieved by the implemented readout method. 

For comparison, the synchronous multi-cycle counting baseline first reshaped the sinusoidal resonator output into a square-wave signal and then performed edge counting using a 50 MHz reference clock. Both methods used the same prototype accelerometer, analog frontend, test period, 1 kHz output update rate, and 1 s averaging before scale-factor and zero-bias evaluation.

**Figure 9:**
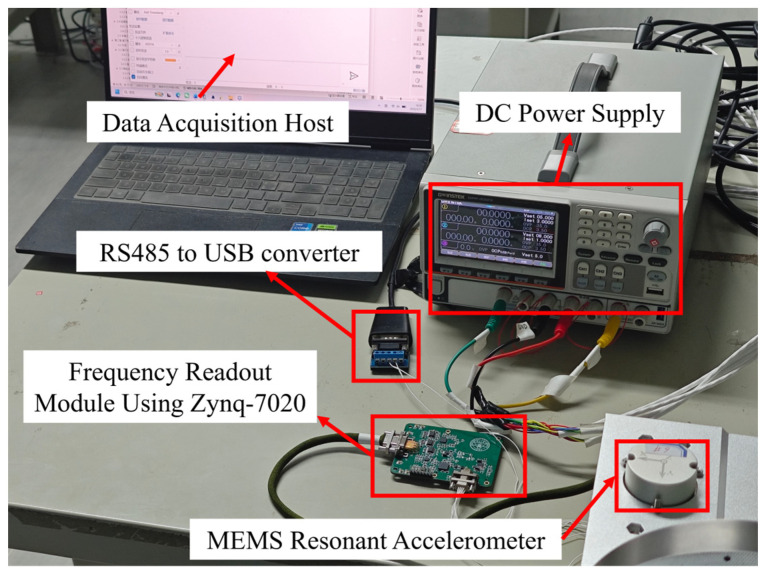
Photograph of the accelerometer performance test platform.

Room-temperature scale-factor tests were carried out by fixing the prototype accelerometer on a precision rotary indexing fixture and rotating it between the +1 g and −1 g positions, as is shown in Figure 9. After a 1 min warm-up period, the output at each position was recorded for 60 s, and the average over the middle 30 s interval was used for scale-factor calculation. For consistency with the subsequent stability evaluation, the 1000 frequency-difference points obtained in each second were first averaged into one point before metric calculation. The room-temperature scale factor was defined as

(26) $$K_{1}=\frac{U_{+1g}-U_{-1g}}{2g}$$

where *U*_+1g_ and *U*_−1g_ are the outputs of the accelerometer at +1 g and −1 g, respectively, and g is the gravitational acceleration. To evaluate measurement consistency, seven repeated tests were carried out under one continuous power-on condition, and the scale-factor repeatability was quantified by the relative standard deviation:

(27) $$K_{1\mathrm{stab}}=\frac{1}{{\bar{K}}_{1}}{\left[\frac{1}{n-1}\sum_{m=1}^{n}{\left(K_{1m}-{\bar{K}}_{1}\right)}^{2}\right]}^{1/2}$$

where $K_{1m}$ is the scale factor obtained in the *m*-th test, and ${\bar{K}}_{1}$ is the mean of the repeated measurements. A smaller $K_{1\mathrm{stab}}$ indicates better scale-factor repeatability. The room-temperature scale-factor test results of the prototype accelerometer are summarized in Table 4.

### 3.4. Room-Temperature Zero-Bias Stability Results

Room-temperature zero-input tests were carried out with the sensitive axis kept horizontal. After power-on, the accelerometer output was continuously recorded for 5400 s at an output update rate of 1 kHz. In this study, zero-bias stability was evaluated over the interval from 1800 s to 5400 s after power-on, corresponding to a 30 min startup condition. For consistency with the room-temperature scale-factor evaluation, the 1000 frequency-difference samples acquired in each second were averaged into one point before the zero-bias metrics were calculated. The measured frequency-difference output was then converted into equivalent acceleration using the mean scale factors obtained from the room-temperature scale-factor tests, i.e., 243.6690045 Hz/g for the synchronous multi-cycle counting method and 243.6693048 Hz/g for the digital closed-loop frequency tracking method.

Let Δ*f*[*m*] denote the averaged frequency difference in the *m*-th second and K denote the corresponding scale factor. The equivalent zero-bias output can be written as

(28) $$a[m]=\frac{\Delta f[m]}{K}$$

The mean zero-bias output over an interval containing N-averaged samples is

(29) $${\bar{a}}_{0}=\frac{1}{N}\sum_{m=1}^{N}a[m]$$

and the zero-bias stability is evaluated by the standard deviation:

(30) $$\sigma_{a,0}=\sqrt{\frac{1}{N-1}\sum_{m=1}^{N}{\left(a[m]-{\bar{a}}_{0}\right)}^{2}}$$

**Figure 10:**
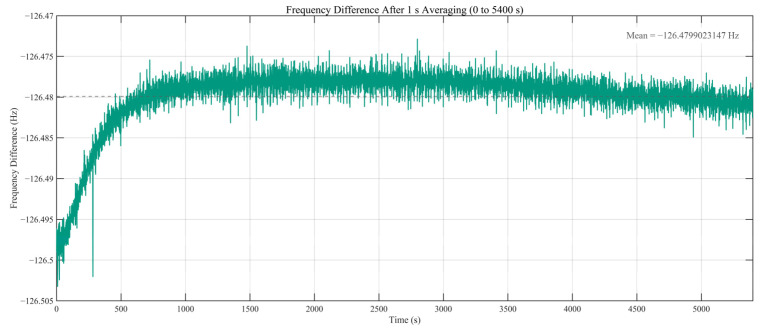
Complete room-temperature zero-bias frequency-difference output from power-on using the synchronous multi-cycle counting method.

**Figure 11:**
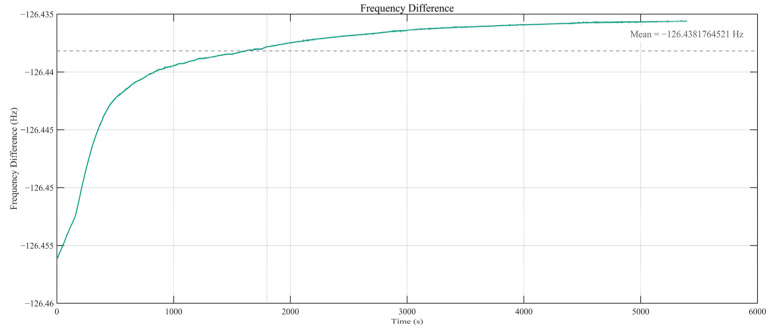
Complete room-temperature zero-bias frequency-difference output from power-on using the proposed digital closed-loop frequency tracking method.

Figure 10 and Figure 11 show the zero-bias frequency-difference outputs from power-on obtained using the synchronous multi-cycle counting method and the proposed digital closed-loop frequency tracking method, respectively. The power-on interval contains a non-stationary output variation before the system gradually approaches a more stable level. This early-stage variation should not be interpreted as the steady-state readout performance of either method. Compared with the synchronous multi-cycle counting result, the phase-difference output exhibits smaller short-term fluctuations under the same room-temperature test condition, while the main quantitative zero-bias evaluation is still performed after the predefined 30 min startup interval.

After data acquisition, the interval from 1800 s to 5400 s after power-on was selected for zero-bias stability evaluation, corresponding to the 30 min startup condition considered in this study. This interval contained 3,600,000 consecutive frequency-difference samples at a 1 kHz sampling rate. For consistency with the scale-factor evaluation, the 1000 samples acquired every second were averaged into one point, yielding 3600 averaged data points for subsequent calculation. The 0–30 min power-on curves therefore reflect the combined thermal stabilization process of the resonator, drive circuit, analog frontend, and readout board rather than the convergence time of the digital FLL–PLL loop alone. After each test, the system was powered off, followed by a 30 min cooling period before the next trial. The zero-bias stability results after a 30 min warm-up under a 1 s averaging window are summarized in Table 5.

To further compare the short-term frequency fluctuation characteristics of the two methods under high-update-rate conditions, the 1 kHz raw frequency-output data were processed using three averaging windows of 1 ms, 10 ms, and 100 ms. The 1 ms case corresponds to the original 1 kHz output, while the 10 ms and 100 ms cases represent short-window averaged outputs. For each averaging window, the standard deviation and peak-to-peak value of the frequency-difference output were calculated, as summarized in Table 6.

These results indicate that the proposed digital closed-loop frequency tracking method suppresses short-term frequency fluctuations more effectively than synchronous multi-cycle counting under high-update-rate conditions. As the averaging window increases, the fluctuation of both methods decreases, but the proposed method maintains lower standard deviations and peak-to-peak variations. To further evaluate long-term stochastic characteristics, Allan analysis was performed on the zero-bias data, and the extracted bias instability and velocity random walk are summarized in Table 7.

Table 7 shows that the proposed digital closed-loop frequency tracking method reduces the average bias instability from 8.023 to 4.720 μg and the velocity random walk from 9.30 × 10^−6^ to 6.48 × 10^−6^ g/√s, indicating improved long-term bias stability and lower stochastic noise under the same room-temperature zero-bias condition.

For a normalized comparison, two relative indicators were calculated using the nominal resonant frequency of approximately 33 kHz as the reference. The first indicator is the relative quantization scale, defined as the theoretical quantization-limited frequency resolution divided by the nominal resonant frequency. At a 1 kHz update rate, this value is 7.36 × 10^−8^ for the proposed digital closed-loop frequency tracking method and 2.00 × 10^−5^ for the synchronous multi-cycle counting method. The second indicator is the relative frequency fluctuation, defined as the measured frequency-difference peak-to-peak value divided by the nominal resonant frequency. Based on the 1-second-averaged steady-state results in Table 5, this value is reduced from 1.13 × 10^−6^ for synchronous multi-cycle counting to 7.30 × 10^−7^ for the proposed method. These two normalized quantities characterize different aspects of the readout performance: The former reflects the theoretical single-update quantization scale, whereas the latter reflects the measured fluctuation range after the specified averaging procedure. Therefore, the normalized comparison should be interpreted as a combined indication of the different quantization mechanisms and the measured steady-state fluctuation behavior rather than as a direct one-to-one comparison between theoretical quantization error and experimental output noise. 

## 4. Discussion

The results support the view that, under the tested high-update-rate conditions, gate-boundary quantization is a major limitation of counting-based readout, while the proposed phase-difference scheme shifts the dominant readout bottleneck toward the phase-extraction chain. This interpretation is consistent with the theoretical comparison in Section 3.1, where the proposed method showed a lower quantization-limited floor than the synchronous multi-cycle counting method at 1 kHz. A high-update-rate FPGA implementation was established with synchronized I/Q processing, valid-boundary alignment, cooperative FLL–PLL tracking, and fixed-point hardware realization.

As summarized in Table 8, existing frequency-measurement and resonant-accelerometer readout methods differ mainly in signal interface and error mechanism. The period-counting-based method in [13] and the FPGA fast-relocking method in [16] improve output-rate regularity or relocking capability, but their frequency information is still obtained after square-wave reshaping and period/counting operations. The CORDIC–differential Kalman method in [17] is a general high-precision algorithm for sampled broadband signals rather than a resonant-accelerometer closed-loop readout scheme. The digital control-loop readout in [18] processes sinusoidal resonator signals with synchronous demodulation and a digital PLL, but its update rate is limited by the microcontroller-based implementation.

This difference in readout mechanism is reflected in the zero-bias output comparison in Figure 10 and Figure 11. Under the same room-temperature conditions, synchronous multi-cycle counting exhibits stronger spike-like fluctuations, mainly because comparator-based waveform shaping makes the readout sensitive to zero-crossing jitter and gate-boundary timing uncertainty. These effects can cause discrete count variations within short update intervals. In contrast, the proposed method derives frequency from continuous phase evolution after quadrature demodulation, low-pass filtering, and CORDIC-based phase extraction, while the finite-bandwidth FLL–PLL loop constrains DDS control word updates. Therefore, its smoother output reflects continuous phase-increment estimation rather than simple numerical smoothing.

The zero-bias results should be interpreted from the viewpoint of power-on stabilization and readout-induced fluctuation. During the early power-on interval, the measured frequency difference contains coupled contributions from the resonator, drive circuit, analog frontend, power supply, and readout board. Because no independent temperature record was acquired, this transient response cannot be assigned solely to thermal drift. Therefore, the post-30 min interval was selected to separate the steady-state readout fluctuation from the hardware stabilization process. In this interval, the smaller fluctuation observed with the proposed method can be explained by the combined effect of continuous phase-increment estimation, band-limited I/Q preprocessing, and finite-bandwidth loop updating, which reduces the influence of discrete edge-count variation and zero-crossing jitter sensitivity associated with waveform-reshaping-based counting. Nevertheless, the residual zero-bias fluctuation is still jointly limited by frontend noise, residual environmental drift, clock stability, phase-extraction noise, and fixed-point implementation errors.

In addition, the 400 Hz initial-offset simulation verifies the digital acquisition and convergence behavior of the FLL–PLL cooperative loop, but it should not be interpreted as a direct validation under shock, broadband vibration, or high-acceleration-gradient conditions. Under dynamic mechanical excitation, the trackability of the loop is jointly determined by the PI-controlled PLL response, FLL smoothing, state-transition thresholds, and DDS frequency-word update process at the 1 kHz update rate. Therefore, the present results demonstrate high-update-rate acquisition and steady tracking under the tested conditions. Further work will focus on equivalent signal-source testing; high-dynamic experimental validation under shock, broadband vibration, and high-acceleration-gradient conditions; adaptive loop-parameter design; and repeated experiments on additional accelerometer prototypes to improve the statistical confidence and generality of the proposed method.

## 5. Conclusions

A high-update-rate frequency readout method for silicon resonant accelerometers was proposed and experimentally evaluated. Instead of estimating frequency through square-wave reshaping and edge counting, the method reconstructs frequency from the phase evolution of the sinusoidal resonant signal through quadrature demodulation, phase extraction, and phase difference. In this way, the dominant readout limitation under high-update-rate operations is shifted from gate-boundary counting uncertainty to the phase-extraction chain, making the method suitable for high-update-rate readout of sinusoidal resonant outputs.

To support acquisition and steady-state tracking in the implemented readout chain, an FLL–PLL-based cooperative loop structure was incorporated. In this implementation, the FLL was used to assist coarse pull-in, while the PLL provided fine adjustment after frequency alignment. This loop organization served as a practical support for the proposed phase-difference readout chain.

The complete scheme was implemented on a Xilinx Zynq-7020 platform and verified through theoretical analysis and prototype experiments. At a 1 kHz update rate, the proposed method showed a lower theoretical quantization limit than the synchronous multi-cycle counting method. In room-temperature experiments, it also provided improved scale-factor stability, and its clearest practical advantage was observed after the startup transients had largely subsided, where the 30 min warm-up zero-bias stability improved from 4.1 μg to 2.4 μg, and the frequency-difference peak-to-peak value decreased from 0.03743 Hz to 0.02410 Hz. These results indicate that the proposed method provides a practical implementation path for high-update-rate readout of sinusoidal resonant signals under the tested conditions.

## Figures and Tables

**Table 1:** Main loop-cooperation and state-switching parameters used in the reported experiments.

Parameter	Meaning	Value/Setting
*α*	Frequency-error smoothing	0.125
*T*_PI_	Pull-in threshold	200 Hz
*T*_S_	SEARCH return threshold	800 Hz
*T*_L_	LOCK entry threshold	0.05 Hz
*T*_U_	Unlock threshold	0.20 Hz
*N*_SEARCH_	Minimum SEARCH dwell	8 update cycles
*N*_LOCK_	LOCK confirmation count	8 update cycles
*β*_PI_	PLL weighting factor in PULL–IN	0.5
*β*_L_	PLL weighting factor in LOCK	0.5

**Table 2:** Quantized SOS coefficients of the 6th-order Butterworth IIR low-pass filter.

Section	b_0	b_1	b_2	a_1	a_2
SOS1	0.000039161271	0.000078322541	0.000039161271	1.975859379030	–0.976015396899
SOS2	0.000039161271	0.000078322541	0.000039161271	1.982228929793	–0.982385450614
SOS3	0.000039161271	0.000078322541	0.000039161271	1.993359026162	–0.993516425839

**Table 3:** Key implementation parameters of the proposed readout system.

Parameter	Value/Setting
ADC sampling rate	500 kHz
Decimation factor	500
Initial DDS/NCO reference	33 kHz
DDS phase width	48 bit
DDS clock frequency	50 MHz
IIR multiplier implementation	54-bit signed multiplier IP core
IIR coefficient format	Signed Q1.52
Phase/frequency update rate	1 kHz
CORDIC input/output width	24 bit/32 bit
CORDIC latency	36 clock cycles

**Table 4:** Scale-factor test results of the prototype accelerometer (Unit: Hz/g).

Test	Synchronous Multi-Cycle Counting Method	Digital Closed-Loop Frequency Tracking Method
1	243.6682138	243.6684908
2	243.6681800	243.6687560
3	243.6685614	243.6690212
4	243.6692662	243.6692865
5	243.6693179	243.6695517
6	243.6693588	243.6698169
7	243.6701337	243.6702107
Average Value	243.6690045	243.6693048
Scale Factor Stability	2.9345 ppm	2.478 ppm

**Table 5:** Zero-bias stability output after 30 min warm-up (1 s averaging window).

Method	Trial	Zero-Bias Stability (μg)	Frequency-Difference Peak-to-Peak (Hz)
Synchronous Multi-Cycle Counting Method	1	4.01774	0.03036
	2	4.53153	0.05531
	3	3.75244	0.02663
	Average	4.10057	0.03743
Digital Closed-Loop Frequency Tracking Method	1	3.97687	0.027392
	2	1.22196	0.021795
	3	2.03777	0.023126
	Average	2.41220	0.02410

**Table 6:** Frequency fluctuation under different averaging windows after a 30 min warm-up.

Averaging Window	Method	Std./Hz	Peak-to-Peak/Hz
1 ms	Synchronous Multi-Cycle Counting Method	8.750 × 10^−1^	2.067
10 ms	Synchronous Multi-Cycle Counting Method	2.802 × 10^−2^	1.390 × 10^−1^
100 ms	Synchronous Multi-Cycle Counting Method	8.378 × 10^−3^	8.429 × 10^−2^
1 ms	Digital Closed-Loop Frequency Tracking Method	1.752 × 10^−3^	9.834 × 10^−2^
10 ms	Digital Closed-Loop Frequency Tracking Method	9.675 × 10^−4^	8.816 × 10^−2^
100 ms	Digital Closed-Loop Frequency Tracking Method	7.650 × 10^−4^	6.970 × 10^−2^

**Table 7:** Allan-analysis-based performance evaluation of the prototype under room-temperature zero-bias conditions.

Method	Trial	Bias Instability, BI (μg)	Velocity Random Walk, VRW (g/√s)
Synchronous multi-cycle counting method	1	7.859	7.54 × 10^−6^
	2	8.869	1.37 × 10^−5^
	3	7.342	6.61 × 10^−6^
	Average	8.023	9.30 × 10^−6^
Digital closed-loop frequency tracking method	1	7.781	6.80 × 10^−6^
	2	2.393	5.41 × 10^−6^
	3	3.989	7.23 × 10^−6^
	Average	4.720	6.48 × 10^−6^

**Table 8:** Comparison with representative high-precision frequency-measurement methods.

Readout Principle	Key Characteristics	Difference from This Work
Period counting + ΔΣ + CIC decimation [13]	Fixed 2 kHz output; low quantization noise	Requires square-wave reshaping; period-domain operation
FPGA closed-loop + Multi-cycle counting [16]	93.4 ms relocking over 975 Hz	Readout coupled to gate time via waveform reshaping
CORDIC–differential– Kalman method [17]	General high-precision measurement for sampled broadband signals	Not designed for resonant-accelerometer closed-loop tracking
ADC + PI control + DAC feedback [18]	Sinusoidal input; 20 Hz output rate	Low update rate limited by microcontroller speed
This work	1 kHz sinusoidal-signal tracking; no reshaping	-

## Data Availability

The data presented in this study are available from the corresponding author upon reasonable request. The FPGA source files and MATLAB scripts used for filter design and offline analysis are also available upon reasonable request.
